# Post Mortem Molecular Biomarkers of Asphyxia: A Literature Review

**DOI:** 10.3390/ijms252111607

**Published:** 2024-10-29

**Authors:** Matteo Antonio Sacco, Isabella Aquila

**Affiliations:** Department of Medical and Surgical Sciences, Institute of Legal Medicine, “Magna Graecia” University, 88100 Catanzaro, Italy; matteoantoniosacco@gmail.com

**Keywords:** asphyxia, biomarkers, molecules, post mortem biochemistry

## Abstract

Asphyxia is a critical condition characterized by inadequate oxygen supply to the body. Post mortem diagnostics of asphyxia present significant challenges in forensic pathology, particularly when there are equivocal signs during autopsy or uncertain circumstantial data. The identification of biochemical biomarkers that indicate asphyxia has emerged as a promising area of research, as these markers can provide vital insights into the physiological changes occurring at the cellular level during asphyxiation. We performed a review of the scientific literature on the search engines Pubmed and Scopus in order to assess the state of the art on this topic. The aim of this study is to analyze which are the most promising markers and methods in the post mortem diagnosis of asphyxia. The literature review highlighted the great potential that molecular investigations can have in the analysis of this type of death, especially considering that hypoxia determines strong biochemical alterations in response to cellular stress. These changes are marked by specific biochemical alterations, which can be detected through various advanced technologies and methodologies, including mass spectrometry, immunohistochemistry, and metabolomic profiling. The review evidenced a combination of markers that can be used for diagnostic purposes in various cases, including mechanical asphyxia, carbon monoxide (CO) poisoning, perinatal asphyxia, and drowning analysis. However, we highlight that, to date, there are still no standard protocols for forensic biochemistry in asphyxia. By scrutinizing the reliability of identified biomarkers and their potential to reshape forensic investigative practices, this research aims to elucidate the critical role that post mortem biochemical analysis can play in diagnosing asphyxia, ultimately contributing to a more nuanced understanding of death-related scenarios and the development of standardized protocols in forensic examinations.

## 1. Introduction

### Definition and Mechanisms of Asphyxia

Asphyxia is a condition arising when the body is deprived of oxygen, leading to unconsciousness and death. It includes various causes and mechanisms, which may depend both on external and internal factors that impede the oxygen supply to vital organs. The pathophysiological mechanisms leading to asphyxia involve a complex interplay of processes resulting in tissue hypoxia. Initially, the lack of oxygen triggers a cascade of cellular events, including anaerobic metabolism, which leads to the accumulation of lactic acid and subsequent metabolic acidosis. This condition can cause cellular damage and organ dysfunction. Additionally, prolonged hypoxia can result in hypoxic–ischemic encephalopathy (HIE) [1].

In forensic cases, asphyxia can result from various common causes, each with distinct characteristics and implications. Some prevalent causes include hanging, suffocation (where external objects obstruct the airways), strangulation (where external pressure on the neck impedes blood and air flow) or choking. Additionally, drowning is a form of asphyxia caused by submersion in water. The post mortem identification of asphyxia often relies on physical external and internal signs such as cyanosis, intense purplish hypostasis, and petechial hemorrhages, which are small blood spots that appear due to capillary rupture [2]. The task of distinguishing asphyxia from other causes of death during post mortem examinations is fraught with challenges, primarily due to the overlapping symptoms and signs that asphyxia shares with other fatal conditions. Forensic pathologists often grapple with the poor specificity of macroscopic signs when diagnosing mechanical asphyxia, making it a complex endeavor. These signs, such as petechial hemorrhages and cyanosis, are not exclusive to asphyxia and may be present in various other conditions. As a result, the determination of asphyxia as a cause of death becomes particularly difficult, especially when these indicators are absent or ambiguous. This complexity is compounded by the need to ascertain whether a death was accidental or intentional, which is crucial for legal and investigative purposes [3]. Therefore, the ability to accurately determine asphyxia as a cause of death remains a pivotal yet challenging aspect of forensic practice.

In recent years, the contribution of post mortem molecular biology in the diagnosis of the cause of death has become increasingly important. In this work, we propose a review of the scientific literature aimed at examining the state of the art regarding the contribution of biomarkers in cadavers as aids in the diagnosis of asphyxia. To this end, we describe the results that have been obtained from scientific studies carried out using experimental models in asphyxia-related deaths, with a focus on suffocation, drowning, and ligature mark vitality.

## 2. Materials and Methods

We performed a review of the scientific literature on the search engines Pubmed and Scopus. For this purpose, we used the following keywords: biomarkers and asphyxia and autopsy; biomarkers and drowning and autopsy; asphyxia and vitality skin ligature. A total of 100 works were obtained from Pubmed, and a total of 62 papers were obtained on Scopus. Subsequently, the authors proceeded to evaluate the title and abstract, after duplicate removal. The works meeting the inclusion criteria were subjected to a reading of the full-paper text. The inclusion criteria included English language and analyses performed on human or animal cadavers by means of molecular investigations. Works that only analyzed the macroscopic autopsy results of asphyxia were removed. Based on the titles and abstracts, a total of 64 papers were selected for full-text reading. A total of 20 papers were included. Another 12 papers were selected using related references in the selected papers.

## 3. Results and Discussion

### 3.1. Key Biochemical Biomarkers in Asphyxia

A molecular biomarker is a characteristic measurable at the molecular level that is used as an indicator of a pathological biological process. Molecular biomarkers can be made of various molecules, including proteins, nucleic acids (DNA, RNA), metabolites, or lipids, and they can be detected and measured in tissues, body fluids (such as blood or urine), or other biological samples. Lactate and pyruvate levels serve as crucial biochemical markers in the post mortem analysis of asphyxia. It is known that during hypoxia, the body shifts from aerobic to anaerobic metabolism, causing a significant rise in lactate levels [4]. This increase can be particularly pronounced in cases of birth asphyxia, where initial lactate levels are useful in assessing the severity of the condition [5]. In neonatal populations, elevated postnatal lactate levels are associated with severe outcomes, indicating potential risks (such as severe disability and death) and aiding in the early detection of complications [6]. Pyruvate, while less studied than lactate, also shows potential as a biomarker due to its involvement in metabolic pathways altered by hypoxia [7]. These values could be subjected to quantification in blood. For example, Fu et al. performed a study in which they investigated the plasma of rats subjected to asphyxiation by CO poisoning and mechanical asphyxiation. The implementation of advanced metabolomic approaches has enabled the identification of candidate biomarkers such as pyruvate, glycerol, and isoleucine, which have demonstrated potential in diagnostics in both cases (CO poisoning and mechanical asphyxia) even if a dependence on the PMI of these markers was observed. Hypoxanthine concentration is another significant biomarker in identifying CO asphyxia. [7]. The application of hypoxanthine as a marker is supported by evidence showing its increased levels in instances of carbon monoxide poisoning, where it serves as a probable biomarker alongside others like 2,3-butanediol [7]. Also, Locci et al. investigated two different models of death in animals, namely ventricular fibrillation and asphyxia. The authors demonstrated how metabolomics using minute-by-minute plasma analysis helps identify pre-arrest asphyxial changes related to the cellular effects of mechanical asphyxia [8].

Additionally, prolonged asphyxia is associated with a measurable increase in alveolar macrophages and mast cells, marked by the hypoxic release of tryptase [9]. The authors measured tryptase levels in femoral blood from two groups: one involving suffocation cases and the other involving sudden cardiac deaths. They found higher tryptase levels in the suffocation group (16.72  ±  4.7 μg/L), but this increase was not statistically significant, likely due to the small sample size (only nine cases in each group). No macroscopical changes in the lungs were visible in the two groups. The differences in the tryptase levels suggest that the absence of oxygen causes an increase in mast perivascular cells with degranulation, presumably related to a stage of inflammation.

Brain tissue biomarkers are critical indicators of hypoxia in forensic pathology. Immunohistochemical analysis has proven to be a robust method for diagnosing acute cerebral hypoxia and ischemia [10]. Specifically, biomarkers such as ubiquitin C-terminal hydrolase L1 (UCHL1) and S100 calcium-binding protein B (S100B) have been identified as effective in determining hypoxic–ischemic encephalopathy, especially in veterinary studies involving calves [11]. Ok et al. investigated the serum concentration levels of brain-related biomarkers in a group of 25 calves with brain damage related to perinatal asphyxia. In addition, brain histopathological and immunohistochemical investigations were performed on 13 non-surviving calves [11]. The authors reported that UCHL1 and S100 were significantly higher in the perinatal asphyxia group compared to controls. UCHL1 is a soluble neuronal protein that exerts protective functions in the brain. It constitutes approximately 1–2% of the proteins present in neurons. Alterations in UCHL1 have been associated with Parkinson’s and Alzheimer’s disease. S100-calcium-binding protein-B is a protein expressed by astrocytes that can act in neurite extension, astrocytosis, and axial proliferation, as well as the inhibition of PKC-mediated phosphorylation. Alterations of this gene are associated with Alzheimer’s disease, Down syndrome, melanoma, and schwannoma. Furthermore, neuron-specific enolase and S100-calcium-binding protein-B are potential biomarkers since their levels are correlated with unfavorable outcomes in hypoxic–ischemic conditions [12].

Research has highlighted stanniocalcin-2 (STC2) as a significant marker, showing down-regulation in mechanical asphyxia samples regardless of the post mortem interval (PMI), age, or temperature [13]. Hu et al. analyzed 12 human brain samples in cases of mechanical asphyxia and found a significant downregulation of this protein compared to controls. Subsequently, cell culture experiments were performed, which indicated that ROS levels after 15 min of hypoxia induce the downregulation of STC2 [13]. The STC2 protein is associated with some oxygen-related functions including the response to oxidative stress. STC2 belongs to the stanniocalcin family and encodes a stanniocalcin-related protein involved in the regulation of intracellular calcium transport and calcium–phosphorus homeostasis. In addition, previous studies have evaluated how neurons expressing STC2 show in vitro resistance to hypoxia. Furthermore, this protein has been correlated with endoplasmic reticulum stress. This consistency across variables underscores the reliability of STC2 as a biomarker for hypoxic brain damage.

Similarly, histopathological studies have underscored the importance of changes in pulmonary tissue, highlighting the role of tissue-specific alterations as indicators of asphyxia [14]. These markers can be independent of external factors such as age, environmental temperature, and the post mortem interval, making them robust indicators of mechanical asphyxia [15]. The application of these markers in forensic practice is advancing, with ongoing research striving to enhance the specificity and reliability of these diagnostic tools. The identification of these markers not only aids in confirming the occurrence of mechanical asphyxia but also helps in differentiating it from other causes of death. In lung tissue, evidence of respiratory failure can be discerned through specific biomarkers that react distinctively to asphyxia. The study of lung tissue components, particularly vessels and alveoli, under asphyxic conditions has highlighted the significance of certain markers [16]. Cecchi et al. analyzed lungs in 62 autopsy cases, of which 34 were mechanical asphyxias, in which they confirmed the presence of HIF1-α in pulmonary vessels [14]. Hypoxia-inducible transcription factor alpha (HIF-alpha) is the central switch that allows cells to act in response to oxygen deficiency. It is a protein of about 800 amino acids with parts that perform different functions. The HIF signaling cascade determines various effects between preventing cells from differentiating and facilitating the formation of blood vessels, promoting wound healing. Strunk et al. evaluated different groups of asphyxias according to the suffocation time and used immunohistochemical investigations with the following antibodies: CD 68, MRP 8, MRP 14 and NP 57 [17]. The analyses demonstrated a significantly higher group of giant cells with early-stage macrophages in cases of prolonged asphyxia. This highlights how the duration of the agony period in asphyxias is associated with the production and proliferation of alveolar macrophages and giant cells at the pulmonary level associated with the timing for the inflammatory reaction to be generated.

Fourier transform infrared spectroscopy has been employed to differentiate asphyxia from sudden cardiac death by analyzing lung tissues in both rats and humans [18]. Zhang et al. subjected a total of 40 samples from 20 rats (10 of which died from asphyxia and 10 died from SCD) and 16 samples from real cases to investigation with FTIR spectroscopy [18]. The authors found biochemical differences between the two causes of death related to alterations in proteins, lipids, and nucleic acids. Furthermore, the authors identified nine different spectra that allowed to discriminate the two causes of death.

Several studies have analyzed multiple biological matrices, i.e., the heart and lungs.

The cytoplasmic upregulation of proteins such as Cytochrome c (Cyto c) and Apoptosis-Inducing Factor (AIF) has been identified as a marker of mechanical asphyxia death [19]. These biomarkers reflect cellular responses to oxygen deprivation. Zhang et al. have, in fact, reported how these two markers located in the mitochondria are transferred to the cytoplasm under hypoxia to initiate the apoptosis process [19]. The authors developed two models to study mechanical asphyxia. One is an animal model where they examined male rats subjected to hanging and suffocation, taking heart and brain samples for analysis. They also analyzed human forensic cases involving mechanical asphyxia (eight cases). Their findings showed an increase in Cytochrome c (Cyto c) and Apoptosis Inducing Factor (AIF) in the heart and brain tissues, suggesting these proteins may indicate stress during asphyxia [20]. The researchers also investigated specific molecular markers, including DUSP1, KCNJ2, miR-122, and miR-3185, in human cardiac samples from asphyxia cases. They found that the increased expression of DUSP1 and KCNJ2 could serve as reliable biomarkers for death caused by asphyxia [20]. DUSP1, a gene that responds to low oxygen levels, reduces ATP production, while KCNJ2 is involved in maintaining cellular activity through its role as a potassium channel. miR-122 helps regulate glucose usage in cells, and miR-3185 is linked to pathways involved in tumor development. Importantly, the authors emphasize that RNA analysis is quick and measurable, making it a promising tool for forensic investigations. In a related study, Ishikawa and colleagues explored hormone levels in 116 autopsy cases from various causes of death. They found that growth hormone (GH) levels in the cerebrospinal fluid were significantly higher in fire-related fatalities with high carbon monoxide (COHb) levels. This suggests that stress responses, triggered by the hypothalamic–pituitary axis, may play a role in these deaths [21].

New biochemical markers, including the NRBC count, IL6, IL1β, PAB, and HSP 70, have also shown promise in diagnosing asphyxia in infants through umbilical serum. These markers could be further explored in post mortem cases to assess their broader applicability [22]. This review highlights that the search for asphyxia markers is closely tied to the body’s inflammatory response and the cellular stress caused by oxygen deprivation. It suggests that certain time-dependent factors, such as the duration of agony and the onset of mechanical asphyxia, are important in understanding cardiac arrest. The authors stress the potential of biological fluid markers, which may show changes more quickly than tissue samples, especially in cases of rapid asphyxia. This underscores the importance of expanding research on both biological fluids and tissues in cases of asphyxia.

### 3.2. Analysis of Ligature Mark Vitality

Histopathology plays a vital role in forensic investigations by helping identify whether ligature marks were made while the person was still alive. This is achieved by examining tissue samples for biological responses that show signs of life, such as the presence of vesicles (small blisters) or the detachment of the skin’s outer layer, the stratum corneum [23]. However, this type of analysis can be challenging, particularly when examining finer details, like in cases involving mole grooves. To improve accuracy, immunohistochemical markers are used. These markers help forensic experts determine if the injury occurred before or after death, which is crucial for understanding the circumstances of the death.

One key marker in ligature marks is aquaporin 3 (AQP3), a protein found in the skin that helps transport water and maintain hydration. AQP3 is located in the deeper layers of the epidermis and is especially useful in cases of hanging or strangulation, where the constriction of the neck leads to dehydration and skin abrasions [24]. The presence of AQP3 in these cases can be an important indicator that the ligature mark was created while the victim was still alive.

Another important marker is fibronectin, which tends to be reduced in ligature marks compared to bruises (ecchymoses). This difference is particularly noticeable in hanging cases, further highlighting the usefulness of histopathological markers in forensic analysis. Additional immunohistochemical markers, such as P-selectin and cathepsin D, help further differentiate between injuries sustained before and after death by assessing wound viability [25].

Markers like tryptase, CD15, and IL-15 are also used to determine whether ligature marks occurred while the person was alive. These markers are associated with the body’s early inflammatory response and blood clotting, and their presence suggests the injury was inflicted during life. For instance, high levels of IL-15 in neck skin indicate a vital reaction, as this protein activates immune cells in response to injury [26,27].

The use of immunohistochemical analysis provides a precise way to understand the biological processes involved, making forensic evaluations more accurate. Therefore, incorporating this type of analysis, especially in uncertain cases, can improve the assessment of ligature marks.

### 3.3. Biomarkers of Drowning

Miyazato et al. studied mRNA levels in lung samples from drowning victims and compared them with other types of asphyxia and causes of death [28]. They found that in drowning cases, the levels of TNF-α, IL-1β, and IL-10 mRNA were significantly higher, suggesting that certain molecular patterns are activated in drowning, particularly early-phase markers of inflammation, which could be useful for diagnosis [28].

Electrolyte imbalances are also crucial in post mortem analyses of drowning cases. In freshwater drownings, a dilution effect occurs, leading to a decrease in serum sodium (Na) and chloride (Cl) levels as water enters the bloodstream [29]. In contrast, saltwater drownings cause hypernatremia (elevated sodium levels) because the high sodium content in the water increases the amount of sodium in the bloodstream [30]. These differences help forensic experts distinguish between freshwater and saltwater drowning. Additionally, altered calcium (Ca) and magnesium (Mg) levels in saltwater drowning cases further aid in this distinction, as these minerals tend to increase after death due to the composition of saltwater. Maeda et al. analyzed these changes by examining serum and pericardial fluid from 56 drowning cases and compared them to sudden cardiac deaths [29]. Also, alterations in blood gases provide another layer of biochemical evidence in drowning investigations. The inhalation of water disrupts normal respiratory function, leading to characteristic changes in the body’s blood gas composition. In cases of drowning, there is often a decrease in blood oxygen levels (hypoxemia) due to impaired gas exchange in the lungs [30]. This is accompanied by an increase in carbon dioxide levels (hypercapnia) as the body fails to eliminate CO_2_ effectively. The resulting respiratory acidosis is a critical indicator of drowning, as it highlights the body’s inability to maintain normal pH levels due to compromised ventilation.

The ability to differentiate drowning from other causes is crucial, particularly in cases where external signs of drowning are not apparent. This differentiation is achieved by examining the molecular alterations in tissues and fluids, which can be indicative of the victim’s exposure to water. The use of post mortem biomarkers, therefore, enhances the accuracy and reliability of post mortem investigations in suspected drowning cases.

Changes in pulmonary surfactant levels are another significant biochemical marker in drowning cases, reflecting the impact of water entry on lung function. Pulmonary surfactants, particularly surfactant proteins A and D (SP-A and SP-D), are critical for maintaining lung stability and function by reducing surface tension in the alveoli [30]. In drowning, the levels of these proteins in the cardiac blood can rise significantly, especially in cases involving secondary pulmonary damage or acute respiratory distress syndrome (ARDS) following trauma. Quan et al. investigated the levels of these two proteins in post mortem serum in a total of 679 cases. The authors considered that the levels of these two proteins were high in both right and left cardiac blood in drowning and ARDS [31]. These results suggest that this increase in surfactant proteins is indicative of the lung’s response to water aspiration and the resultant alveolar damage. Recent proteomic analyses have highlighted two significant biomarkers, apolipoprotein A1 and α-1 antitrypsin, which are instrumental in distinguishing drowning from other causes of death [32]. Hernández-Romero et al. measured the levels of these proteins in plasma in 25 autopsy cases, of which 16 died by drowning [32]. In particular, ApoA1 showed higher levels in drowning while α-1 antitrypsin showed lower levels [32]. ApoA1 is a protein with important roles in several respiratory diseases, and it has a role in controlling inflammation and oxygen stress. Instead, a decrease in α-1 antitrypsin is associated with destruction of the alveolar wall as well as emphysematous pulmonary alterations. These biomarkers are characterized by their unique expression patterns in drowning cases, offering forensic scientists valuable insights into the physiological changes that occur during drowning events.

The utilization of strontium and diatom tests as indicators of drowning has emerged as an effective method in forensic investigations. Blood strontium levels, for instance, serve as a valuable marker for diagnosing drowning, as they tend to be elevated due to the mineral composition of the water [10]. Pérez-Cárceles investigated 67 drowning cases (of which 53 occurred in saltwater and 14 in freshwater) with analyses of the levels of different concentrations of Sr, Mg, Na, Cl, Ca, and Fe in right ventricular blood, left ventricular blood, and peripheral blood [33]. Analyses confirmed that strontium is the best serum parameter for the analysis of seawater drowning. In the case of freshwater downing, this value must be combined with other markers including iron to increase diagnostic sensitivity.

Aquaporins play a critical role in the body’s response to drowning by regulating water transport across cell membranes [34]. These water channel proteins help maintain osmotic balance by facilitating the movement of water in and out of cells, thus contributing to cellular hydration and overall fluid homeostasis [35]. In the context of drowning, the expression of aquaporins can be altered, impacting the body’s ability to manage the sudden influx of water. Forensic studies have shown differential expression of aquaporins, such as AQP5 and AQP2, in tissues affected by drowning, which could provide insights into the underlying physiological changes [36,37].

### 3.4. Practical Applications and Implications in Forensic Medicine

Although, traditionally, the diagnosis of asphyxia in forensic pathology is based on autopsy and the microscopic histological analysis of tissues, we suggest integrating the diagnosis with the qualitative or quantitative analysis of cited biomarkers. The scientific literature has, in fact, highlighted numerous molecular biomarkers that may prove promising for the diagnosis. This evaluation could prove to be a support for diagnosis, especially in dubious cases or with uncertain data. To date, there is not a single biomarker which could be considered better than others for sensibility and specificity, but, according to the specific case, we suggest the following for diagnostic purposes:

- In the case of mechanical asphyxia, we highlight the usefulness of immunostaining, Western blotting, ELISA, or mRNA analysis investigations on one or more tissue samples described in Table 1, depending on the specific case;
- In the case of analyses of the vitality of the ligature mark, we suggest immunostaining on neck skin samples according to the markers described in Table 2;
- In the case of drowning, we emphasize the usefulness of aquaporins and the investigations described in Table 3 for the differential diagnosis between saltwater and freshwater drowning.

The analysis of these markers may, however, present some limitations. One major difficulty is the lack of standardized cut-off values for many biomarkers; without these reference points, it becomes challenging to draw definitive conclusions from only the biochemical data [38]. Additionally, post mortem changes such as autolysis and putrefaction can alter the levels of various markers, potentially leading to misleading results. For instance, for several described markers, their levels can fluctuate due to post mortem changes, complicating their interpretation. This variability requires careful consideration and corroboration with other clinical and forensic evidence to ensure accurate conclusions. Furthermore, it should be considered that these markers may be affected by some variables, such as survival time, but also by sharing similar levels with other causes of death. [39]. The interpretation of some biochemical results may require further studies, since some detected markers are also present in non-asphyxial conditions (for example, HIF-1α). The complex nature of biochemical processes after death adds another layer of complexity, as various factors such as temperature, humidity, and body condition can affect biomarker stability and detection. These challenges underscore the need for advanced methodologies and technologies to improve the identification and validation of asphyxial biomarkers [40]. Therefore, we highlight the importance of continuing research in this area with new experimental models that include a greater number of cases in order to overcome these limits. Furthermore, we emphasize that the combination of multiple markers could increase the diagnostic sensitivity of molecular investigations and facilitate data interpretation. Clearly, the choice of markers to be investigated depends on the specific case and must take into account the available samples and the economic and logistical resources for the assessment. In this regard, to date, there are no guidelines in forensic pathology that require the performance of biomolecular investigations in cases of suspected asphyxia. However, we highlight the usefulness of these analyses with standardized protocols in post mortem biomarker analysis, which would help establish consistency and reliability in forensic investigations. Standardization would involve creating uniform guidelines for sample collection, storage, and analysis, thereby reducing variability and improving the reproducibility of results. Moreover, the establishment of standardized protocols would facilitate the comparison of findings across different studies, promoting collaboration and knowledge sharing among researchers in the field. As the field progresses, the implementation of standardized protocols will be crucial in advancing the accuracy and reliability of post mortem biomarker analysis for mechanical asphyxia.

## 4. Conclusions

This literature review has proved the usefulness of a combination of biomarkers in different cases, including mechanical asphyxia, perinatal asphyxia, CO poisoning, ligature mark analysis, and drowning. Therefore, today, the forensic pathologist has a wide range of available biomarkers that can be analyzed in different samples and with different methods. These analyses can be supportive for diagnostics of complex or uncertain cases of asphyxia. Future directions of research will concern a greater application of these methods in post mortem diagnostics with greater standardization and an increase in research in this area. Future research will have to evaluate a larger case series in the near future and seek a combination of markers that is not affected by variables such as the PMI or the overlap with other causes of death, in order to improve the degree of diagnostic sensitivity and cost containment. As the field continues to evolve, ongoing research will be critical in identifying and validating these biomarkers, ultimately improving the efficacy of forensic investigations.

## Figures and Tables

**Table 1:** Summary of asphyxia markers from literature review.

Biological Sample	Asphyxia Markers	Function	Type of Asphyxia	Method for Investigation
Blood	Lactate	Metabolite	Perinatal asphyxia	Biochemical assay
	Pyruvate, glycerol, and isoleucine	Metabolites	Mechanical asphyxia and CO poisoning	GC-MS
	Hypoxantine and 2,3-butanediol	Metabolites	CO poisoning	GC-MS
	Tryptase	Product of degranulation of mast cells	Suffocation	Immunoassay
	UCHL, S100B, NSE	Astrocyte proteins	Hypoxic–ischemic conditions	ELISA
Brain	STC2	Glycoprotein with autocrine or paracrine functions	Mechanical asphyxia	Western blotting and immunofluorescence
	Cyto c and AIF	Apoptosis markers, released from mitochondria	Mechanical asphyxia	Western blotting
Lungs	HIF1-α	Hypoxia-induced transcription factor	Mechanical asphyxia	Immunostaining
	CD 68, MRP 8, MRP 14 and NP 57	Macrophages and giant cells markers	Prolonged asphyxias	Immunostaining
Heart	Cyto c and AIF	Apoptosis markers, released from mitochondria	Mechanical asphyxia	Western blotting
	DUSP1, KCNJ2, miR-122, miR-3185	ER stress-related protein CHOP	Mechanical asphyxia	RNA analysis
Cerebrospinal fluid	GH	Hormone	Markers for CO poisoning	Immunoassay
Umbelical serum	NRBC count, IL6, IL1β, PAB, and HSP 70	Neonatal asphyxia markers	Perinatal asphyxia	ELISA

**Table 2:** Summary of ligature vitality markers from literature review.

Biological Sample	Asphyxia Markers	Function	Type of Asphyxia	Method for Investigation
Skin	Aquaporin 3	Water channel protein	Hanging and strangulation	Immunostaining
	P-selectin and cathepsin D	Proteins related to endothelial damage and lysosomal action, respectively	Hanging	Immunostaining
	Tryptase, CD15, and IL-15	Inflammatory markers	Hanging	Immunostaining

**Table 3:** Summary of drowning markers from literature review.

Biological Sample	Asphyxia Markers	Function	Type of Asphyxia	Method for Investigation
Lung	TNF-α, IL-1β, and IL-10 mRNA levels	Early-phase mediators of inflammation	Drowning	mRNA analysis
	RAGE and AQP5	Inflammatory marker and water channel protein	Drowning	mRNA analysis, Western blotting, immunostaining
Pericardial fluid	Na and Cl	Electrolytes	Seawater and freshwater drowning	Ion-selective electrodes method
Blood	SP-A and -D	Pulmonary surfactant-associated proteins	Drowning and secondary pulmonary damage involving	Immunoassay
	ApoA1 α-1 antitrypsin	Proteins involved in various lung diseases	ARDS after traumas Drowning	LC/MS
	Strontium	Metal	Seawater and freshwater drowning	Biochemical analysis
Kidney	AQP2	Water channel protein	Seawater and freshwater drowning	Immunostaining
